# Pickering emulsion loaded with total flavonoids from *Dracocephalum moldavica* L. potentially promotes angiogenesis in the ischemic penumbra after cerebral ischemia reperfusion

**DOI:** 10.3389/fbioe.2025.1702899

**Published:** 2025-10-31

**Authors:** Tianyi Gao, Lu Wu, Yuru Ma, Weikai Zhai, Lu Zhang, Wei Cong, Zhiping Cai, Chengli Cui, Liang Li

**Affiliations:** ^1^ Department of Basic and Forensic Medicine, Baotou Medical College, Baotou, China; ^2^ Xinxiang Central Hospital, Xinxiang, Henan, China; ^3^ Cangzhou People’s Hospital, Cangzhou, Hebei, China

**Keywords:** pickering emulsion, *Dracocephalum moldavica* L., cerebral ischemia-reperfusion, vascular regeneration, ischemic penumbra

## Abstract

**Background:**

Ischemic stroke is a major disease threatening human health. Currently, its therapeutic options are extremely limited, and common thrombolytic therapy tends to cause tissue damage. Traditional Chinese and Ethnic Medicine have a centuries-long clinical history in treating ischemic stroke. *Dracocephalum moldavica* L., a traditional Mongolian herb distributed in China’s Inner Mongolia, is one such example.

**Methods:**

We adopted the ultrasonic extraction method to obtain total flavonoids from *Dracocephalum moldavica* L. (TFDM) and validated its efficacy in an animal model of ischemia-reperfusion injury. A novel Pickering emulsion was used to load TFDM as a new delivery system, and we characterized its key properties such as physical properties and bioavailability.

**Results:**

The extraction efficiency of TFDM obtained by the ultrasonic extraction method was improved, with the extraction rate of the new protocol reaching 65% compared with the traditional method. Subsequent animal model validation confirmed that TFDM extracted by this method had significant therapeutic effects: enhancing vascular perfusion in the ischemic penumbra after reperfusion, reducing cerebral infarct volume, and promoting neurological function recovery. Mechanistically, TFDM upregulated the expression of VEGF, VEGFR2, and CD34 in the ischemic penumbra of cerebral ischemia-reperfusion injured rats post‐injury. Concomitantly, these molecular changes accelerated microvascular regeneration and microcirculation reconstruction in the ischemic penumbra. Further studies have confirmed that the delivery strategy using Pickering emulsion to load TFDM can effectively overcome the inherent drawbacks of TFDM, such as its short storage time and low bioavailability.

**Conclusion:**

Overall, this study provides robust evidence for TFDM’s neuroprotective effects, supporting its potential as a novel therapeutic candidate for stroke prevention and treatment. Notably, TFDM encapsulated in Pickering emulsion is a practical, efficient approach with promising prospects for future stroke therapy.

## 1 Introduction

Ischemic stroke is a malignant cerebrovascular event caused by local cerebrovascular infarction. In China, the incidence of stroke among individuals aged 40 years and above is approximately 0.5%, which is significantly higher than the global average of 0.158% (Jing et al., 2023). Cerebral ischemia-reperfusion injury (CIRI) induced by conventional intravenous thrombolysis has become increasingly prevalent (Zhang et al., 2022). The range of therapeutic agents for ischemic stroke is limited, with only recombinant tissue plasminogen activator (rtPA) being the FDA-approved drug for this indication (Hacke et al., 1995). These aforementioned issues have limited the prognosis and treatment of stroke.

In fact, Traditional Chinese and Ethnic Medicine has a long-standing history of application in the treatment and rehabilitation of patients with ischemic stroke, accompanied by extensive clinical experience. The development and utilization of Traditional Chinese and Ethnic Medicine for stroke management represent a promising and broad-horizon approach. Studies have demonstrated that numerous traditional Chinese (ethnic) medicinal agents, such as *Salvianolic Acid C* (Yang et al., 2021) and *Panax notoginseng saponins* (Hui et al., 2017), can increase vascular density in ischemic regions and promote vascular proliferation.

*Dracocephalum moldavica* L. is distributed in Inner Mongolia, Xinjiang, and other regions of China, with a clinical application history of several hundred years. The chemical constituents of the whole herb of *D. moldavica* L. include volatile oils, steroids, terpenoids, flavonoids, and other components (Jin et al., 2020). TFDM have demonstrated extensive pharmacological activities both *in vivo* and *in vitro*, including cardiovascular protective effects, antioxidant, antibacterial, and antifungal activities (Zhan et al., 2024). In addition, the exertion of TFDM’s biological effects may be associated with the activation of Src kinase in the vascular endothelial growth factor (VEGF) signaling pathway, as well as the PI3K/AKT signaling pathway (Hu et al., 2023a). These pathways play crucial roles in regulating vascular formation and angiogenesis, thereby providing evidence supporting the potential application value of TFDM in promoting angiogenesis for the treatment of ischemic stroke.

Despite the high therapeutic value of TFDM in disease treatment, it is difficult to maintain long-term stability under natural conditions, as it is susceptible to degradation under various environmental factors. Additionally, its oral bioavailability remains low. To address the issues of limited therapeutic agents and inefficient delivery of active components, it is feasible to develop an innovative therapy for stroke patients based on TFDM combined with a modern drug delivery system.

First, we adopted an innovative ultrasonic extraction protocol to extract TFDM and verified the effectiveness of the extracted components. Subsequently, a novel Pickering emulsion was prepared using SSOS, CS, and TA as raw materials, and TFDM was encapsulated within it to resist the degradation of its active components caused by external factors such as light, oxygen, temperature, and pH.

The experimental results showed that TFDM extracted using the new protocol could effectively alleviate reperfusion injury and promote angiogenesis in ischemic regions. Additionally, the bioavailability of TFDM encapsulated in the Pickering emulsion was higher than that of TFDM administered via simple oral administration alone. Our research demonstrates that the therapeutic strategy of delivering TFDM via Pickering emulsion loading holds significant application potential in stroke treatment, thereby providing a novel approach for the management of stroke.

## 2 Materials and methods

### 2.1 Materials

Nylon monofilament sutures (diameter: 0.38 mm) were purchased from Beijing Sinovac Technology Co., Ltd. (Beijing, China); 2,3,5-Triphenyltetrazolium chloride (TTC, cat. no. t8877), toluidine blue, and fluorescein isothiocyanate-dextran (FITC-dextran, cat. no. fd 2000s) were obtained from Sigma-Aldrich (St. Louis, Missouri, United States); Protein extraction kits (cat. no. c1051) and ECL protein luminescence detection kits (cat. no. p1020) were purchased from Beijing ABigen Biotechnology Co., Ltd. (Beijing, China); Antibodies against VEGFA, VEGFR2, and CD34 were obtained from Pyrotek Group (Wuhan, China); BCA protein quantification kits were purchased from Shanghai Aupmei Biotechnology Co., Ltd. (Shanghai, China); Sprague-Dawley (SD) rats were provided by SPF Biotechnology Co., Ltd. (Beijing, China); Universal two-step detection kits (cat. no. pv-9000) were obtained from Zhongshan Jinqiao Biotechnology Co., Ltd. (Beijing, China); SSOS was purchased from Hangzhou Prostar Starch Co., Ltd. (Hangzhou, China); TA and CS were obtained from Shanghai Macklin Biochemical Technology Co., Ltd. (Shanghai, China); Corn oil was purchased from Shandong Xiwang Food Co., Ltd. (Shandong, China). Avertin and sodium pentobarbital are obtained through application to the Laboratory Animal Center of Baotou Medical College.

### 2.2 Methods

#### 2.2.1 Extraction and content analysis of TFDM

*Dracocephalum moldavica* L. was provided by the Medicinal Botanical Garden of Baotou Medical College. The plants were air-dried and subsequently ground into powder under low-temperature conditions. In the experiment, ethanol was used as the extraction solvent, and the extraction was performed in a constant-temperature ultrasonic extractor (Kunshan Ultrasonic KQ-500E model, China, 500 W). In the experiment, ultrasonic disruption extraction was conducted by adjusting the volume percentage of the extraction solvent (ethanol, 30%–90%), ultrasonic time (0.5 ∼ 4 h), temperature (30 °C ∼ 80 °C), and the ratio of powder to solvent (1:20 ∼ 1:80). The extract was concentrated under reduced pressure using a rotary evaporator (Yalong RE-2000A, China) to remove ethanol. The resulting extract was freeze-dried, ground into powder, and stored in a −20 °C refrigerator. The absorbance values of flavonoids under different extraction conditions were measured at a wavelength of 510 nm using an ultraviolet spectrophotometer (Shimadzu UVmini-1240, Japan). The content of TFDM was calculated by comparing these values with a standard solution curve (Matic et al., 2017), where rutin was used as the standard reference substance.

#### 2.2.2 Establishment of the middle cerebral artery occlusion (MACO) model

This experimental protocol was approved by the Animal Ethics and Welfare Committee of Baotou Medical College and conducted in strict accordance with relevant guidelines. The animal ethics approval number is Animal Ethics Review of Baotou Medical College (2023) No. 31. Sprague-Dawley (SD) rats were housed in a constant-temperature environment (22 ∼ 24 °C) with free access to food and water, and their average body weight was 280 ± 30 g. The establishment of the MCAO reperfusion model was performed with reference to methods reported in the literature (Li et al., 2023). During the operation, rats were anesthetized by intraperitoneal injection of Avertin (300 mg/kg, 2.5%w/v solution), the body temperature of the rats was maintained at 37 °C ± 0.5 °C. After skin suturing, the rats were placed in an incubator for proper care. Two hours after surgery, the suture plug was removed to achieve common carotid artery reperfusion. For the sham operation group, no monofilament nylon suture was introduced. After the rats regained consciousness, they were scored according to the Zea-Longa 5-point scoring system (Longa et al., 1989) to evaluate the success of model establishment. Rats with failed model establishment were excluded from subsequent experiments.

#### 2.2.3 Grouping and management of experimental animals

The rats were randomly divided into three groups: 50 rats in the sham operation group (Sham); 160 rats that successfully completed MCAO reperfusion were further divided into two groups: the cerebral ischemia-reperfusion model group (I/R) and the cerebral ischemia-reperfusion model group treated with TFDM (I/R + TFDM), with 80 rats in each group. Each group was further subdivided into four time points: 1 day, 3 days, 7 days, and 14 days after surgery, with 20 rats at each time point. The I/R + TFDM group received intragastric administration of a 50 mg/kg TFDM aqueous solution after surgery, with the treatment administered to conscious rats. The gavage protocol was performed with reference to methods reported in the literature (Jia et al., 2017). Meanwhile, rats in the Sham group and I/R group received intragastric administration of an equal volume of pure water. The administration was performed once every 12 h, continuing until 6 h before sampling.

#### 2.2.4 Preparation of tissue samples

All animals were treated with intraperitoneal injection of excessive sodium pentobarbital before sacrifice (100 mg/kg, 2%w/v solution). For the Sham group, brain tissues were only collected at 1 day post-surgery for TTC staining and Western Blot analysis. For the other groups, animals were sacrificed 6 h after the last gavage on days 1, 3, 7, and 14, and brain tissues were immediately harvested. Brain tissues were immediately subjected to TTC staining. Brain tissues intended for Western Blot analysis were stored at −80 °C. For brain tissues used in Nissl staining and immunohistochemical analysis, they were fixed overnight in 4% paraformaldehyde, followed by paraffin embedding for section preparation.

#### 2.2.5 Evaluation of neurological function

The modified Neurological Severity Score (mNSS) was used to evaluate the severity of injury in rats, including indicators such as balance ability, tactile perception, visual function, abnormal behaviors, muscle tone, sensory function, and motor ability. The scoring results of each group were recorded.

#### 2.2.6 TTC staining

The TTC staining method was used to detect the range of ischemic infarction in the MCAO reperfusion animal model. The staining procedure was performed with reference to the instructions of the staining reagent. The area and total volume of individual infarcted regions were quantified using ImageJ analysis software, and the cerebral infarction volume data were presented as a percentage of the total brain tissue volume.

#### 2.2.7 Nissl staining

Nissl staining enables the staining of Nissl bodies and observation of neuronal damage. The staining procedure was performed with reference to the instructions of the staining reagent. Neuronal damage in the ischemic penumbra region of rats in each group was observed using a 100× microscope (NI-U model, Nikon Corporation, Japan), and images were collected.

#### 2.2.8 FITC-dextran perfusion and image analysis

FITC-labeled dextran was injected into the plasma of animals. Cerebral plasma perfusion, microvascular diameter, and morphological characteristics were observed using a laser confocal microscope (Nikon, Japan). The operation method was performed with reference to the instructions of the experimental reagent. Three-dimensional reconstruction of images was performed using a laser confocal microscope. For all experiments, each sample contained at least 5 replicates, with 5 animals in each group.

#### 2.2.9 Immunohistochemistry

The method for the immunohistochemical experiment was performed following the standard procedures described in the kit instructions. In the experiment, the primary antibodies (mouse anti-rat VEGF protein, mouse anti-rat VEGFR2, and mouse anti-rat CD34) were all used at a dilution of 1:300. In the negative control group, non-immune serum was used to replace the primary antibody. The secondary antibody (rabbit anti-rat IgG) was used at a dilution of 1:500. Experimental specimens were observed under a 400× optical microscope (NI-U model, Nikon, Japan) to examine the ischemic penumbra region in the medial caudate nucleus. Five non-overlapping visual fields were selected for observation, and images were collected to count the number of positive cells. For all experiments, at least 5 sections were analyzed per sample in each group, with 5 animals included in each group.

#### 2.2.10 Western Blot analysis

All tissue protein samples were extracted using a protein extraction kit (Beyotime, China). The preparation of experimental samples and the determination of protein concentration were performed with reference to the kit instructions. In the experiment, the primary antibodies used were as follows: β-tubulin (1 : 2000 dilution), VEGFA (1 : 3000 dilution), VEGFR2 (1:5000 dilution), and CD34 (1:2000 dilution). The secondary antibody used was: horseradish peroxidase (HRP)-conjugated rabbit anti-mouse IgG (1:5000 dilution). Image acquisition and analysis were performed using ImageJ software.

#### 2.2.11 Preparation of ternary complexes

A 0.5% (w/v) CS solution was prepared by dissolving CS in a 1.0% (w/v) acetic acid solution. A 10% SSOS suspension was prepared under water bath conditions at 50 °C. A 0.5% (w/v) TA solution was prepared by dissolving TA in distilled water with stirring until complete dissolution. TA was added to SSOS at a ratio of 1:1, followed by the slow addition of an equal volume of CS solution to prepare the SSOS-CS-TA complex solution.

#### 2.2.12 Preparation of TFDM-Pickering emulsion

TFDM was added to corn oil at a concentration of 0.5% (w/v), and the mixture was blended at 600 r/min for 1 h at 60 °C. The mixture was then mixed with the SSOS-CS-TA complex solution at a ratio of 1:1, followed by high-speed shearing at 15000 r/min for 1 min to obtain the TFDM-Pickering emulsion.

#### 2.2.13 Determination of TFDM-Pickering emulsion properties

The particle size of droplets in the TFDM-Pickering emulsion was determined using a laser particle size analyzer (Mastersizer 3000, United Kingdom). The droplet size distribution of Pickering emulsions with and without TFDM encapsulation was observed using an optical microscope, with images captured at magnifications ranging from ×100 to 400×. The encapsulation efficiency percentage (EE, %) of TFDM was determined using an ultraviolet-visible spectrophotometer, and the measurement method for the emulsion encapsulation efficiency was referenced from the literature (Wang et al., 2023).

#### 2.2.14 Determination of oral bioavailability of TFDM in rats

Twelve 8-week-old SD rats to be tested, with half male and half female and a body weight of 280 ± 30 g, were divided into two groups: the TFDM aqueous solution gavage group (TFDM) and the TFDM-Pickering emulsion gavage group (TFDM-Pickering). Prior to gavage administration, the animals were fasted for 12 h with free access to water. Both groups were administered at a dose of 50 mg/kg. Blood samples were collected from the tail of each rat at 9 time points after gavage (5 min, 15 min, 30 min, 1 h, 2 h, 4 h, 8 h, 12 h, and 24 h). The blood samples in anticoagulant tubes were centrifuged at 5,000 r/min for 10 min, and the upper plasma was separated for subsequent use. The absorbance was measured at 510 nm using a full-spectrum multimode microplate reader (INFINITE 200 PRO, Germany) to calculate the concentration of TFDM in the plasma samples to be tested. A standard solution calibration curve was established using rutin, and the concentration of TFDM in the plasma samples was recorded and analyzed. Calculation of TFDM concentration-time curve in rat plasma: The formula for calculating oral relative bioavailability (Fr) is: Fr = (AUC_P_/AUC_T_) × 100%. In this formula, AUC_P_ represents the area under the concentration-time curve (AUC_0-24_h) in the TFDM-Pickering group, and AUC_T_ represents the AUC_0-24_h in the TFDM group.

### 2.3 Statistical analysis

All data are expressed as mean ± standard deviation ( x ± s ), and each group of experiments was repeated three times. Statistical analysis was performed using one-way analysis of variance (ANOVA) combined with *post hoc* tests, with a *p* < 0.05 considered statistically significant. Statistical graphs were plotted using GraphPad Prism 8.0 software (California, United States).

## 3 Results

### 3.1 Optimization conditions for the extraction of TFDM

**FIGURE 1:**
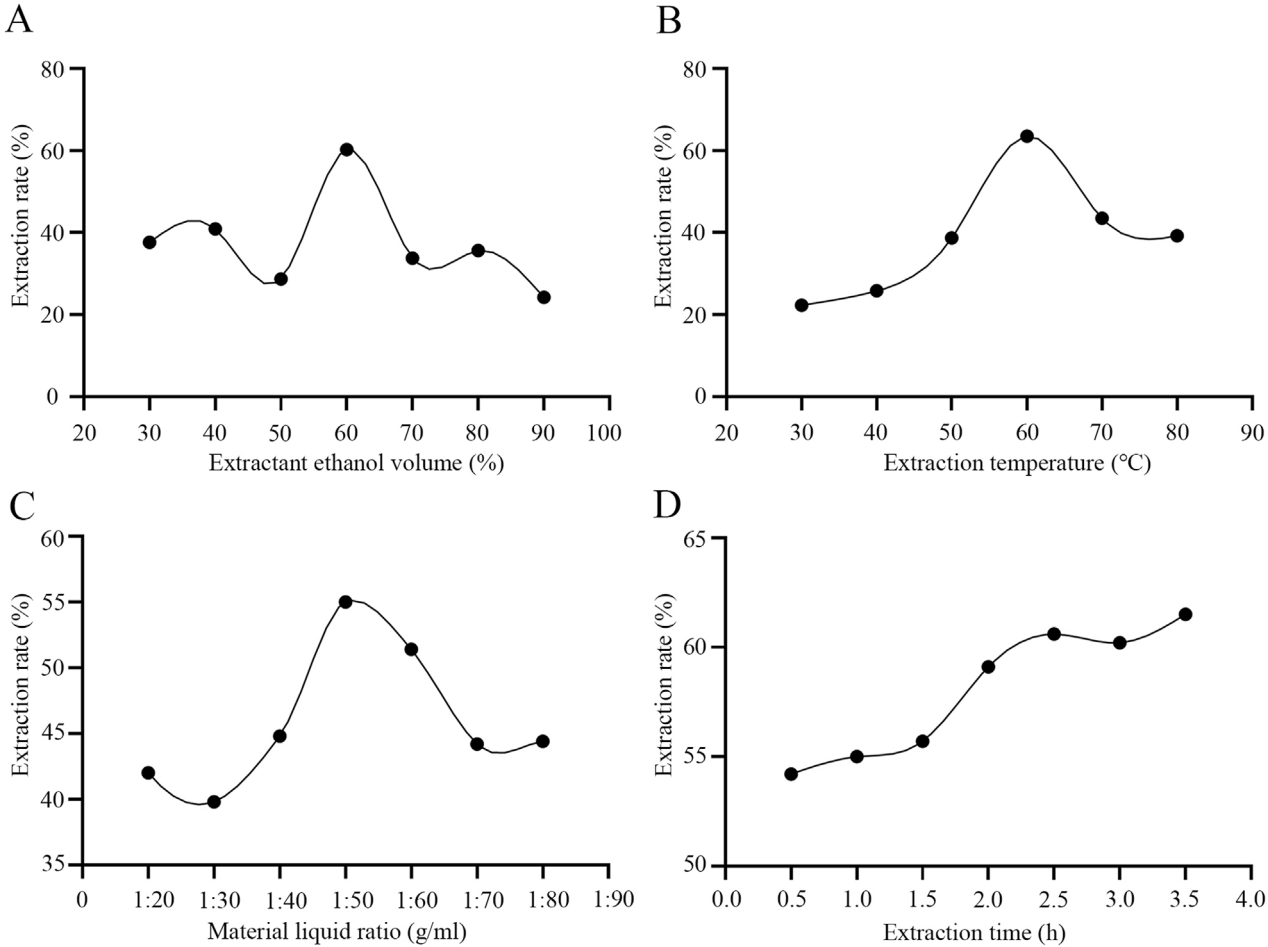
Effects of Extraction Conditions on Total Flavonoids from *Dracocephalum Moldavica* L. **(A)** The optimal detection of ethanol volume percentage. **(B)** The optimal detection of extraction temperature. **(C)** The optimal detection of the volume ratio of *Dracocephalum Moldavica* L. to the extraction solvent. **(D)** The optimal detection of extraction time.

In the traditional soaking extraction method for *D. moldavica* L., water is often used as the extraction solvent, which fails to ensure the extraction efficiency of TFDM. We employed the ultrasonic disruption extraction method using an aqueous ethanol solution as the extraction solvent. By optimizing four key parameters during the extraction process—extraction temperature, ethanol volume percentage, volume ratio of *D. moldavica* L. to the extraction solvent, and extraction time—the extraction efficiency of TFDM was improved. The experimental results showed that under the conditions of a temperature of 62 °C, an ethanol volume percentage of 65%, a volume ratio of extraction solvent to *D. moldavica* L. of 40:1, and an extraction time of 2.5 h, the extraction efficiency of TFDM by the ultrasonic disruption extraction method could reach 65% (Figure 1). The use of the ultrasonic disruption extraction method has improved the yield of TFDM, and the optimized extraction conditions have enabled better preservation of the active substances in TFDM.

### 3.2 The use of TFDM reduces cerebral ischemic infarct volume and improves neurological function in model rats

**FIGURE 2:**
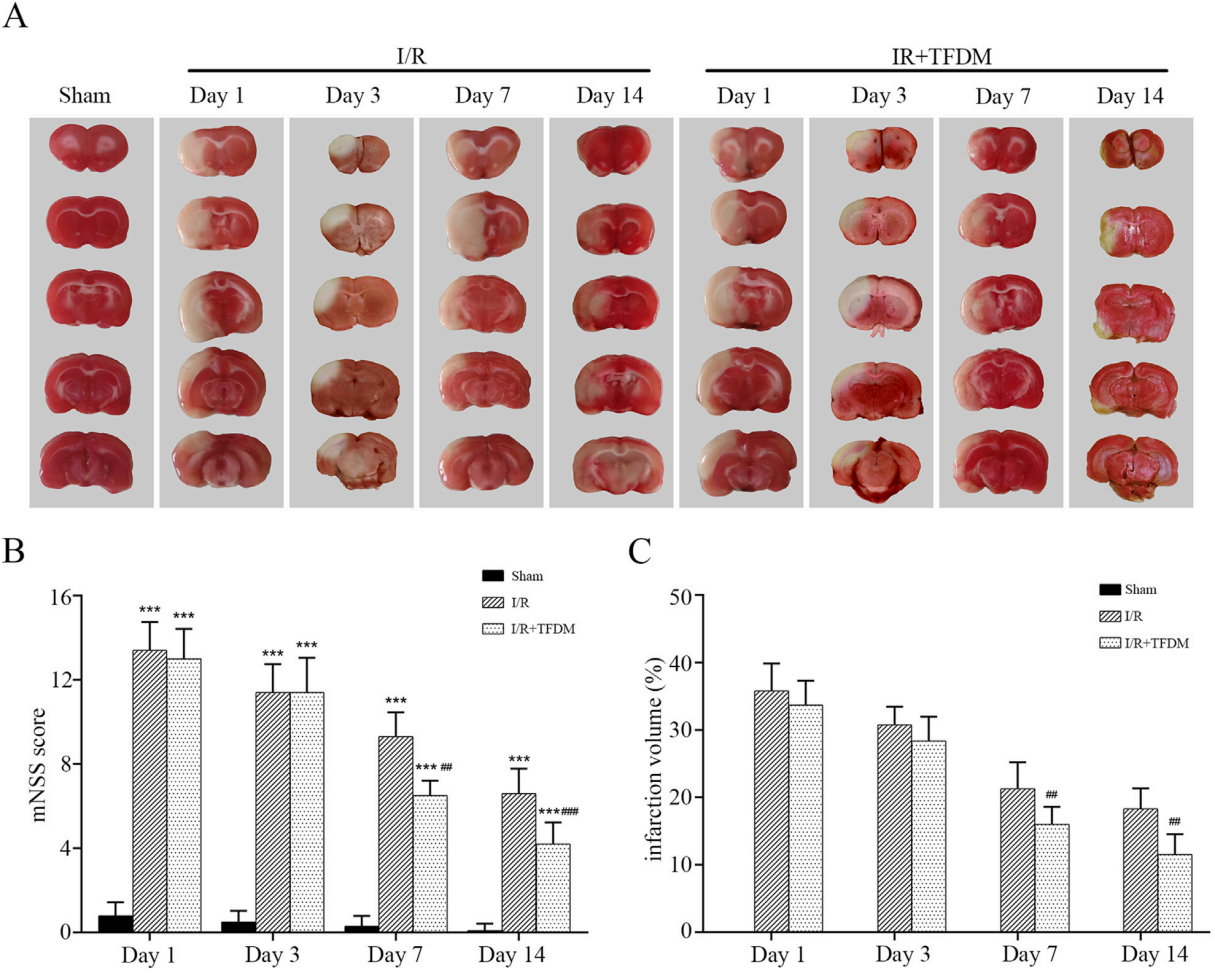
Effects of TFDM on post-treatment cerebral infarction volume and neurological function in rats. **(A)** TTC staining reveals a cerebral infarction image. **(B)** Statistical analysis of mNSS scores. **(C)** Statistical analysis of infarct volume via TTC staining. ^***^ *p* < 0.001 (compared with Sham group), ^##^ *p* < 0.01, ^###^ *p* < 0.001 (compared with I/R group).

We applied TFDM in animals with the MCAO reperfusion model. Image analysis of coronal brain sections from the model animals showed that TFDM significantly reduced the cerebral ischemic infarct volume in MCAO rats (Figure 2A). Temporal analysis showed that the cerebral infarct volume in rats of each group gradually decreased over time. On days 7 and 14, that in rats of the I/R + TFDM group was significantly lower than that in the I/R group (p < 0.01), while there was no statistical significance between the two groups on days 1 and 3 (Figure 2C). To investigate the promotional effect of TFDM on neurological function recovery in MCAO reperfusion rats, we observed the changes in mNSS on days 1, 3, 7, and 14 after surgery. The results showed that the mNSS in both the I/R group and the I/R + TFDM group was significantly higher than that in the sham group at all time points (p < 0.001). However, the scores gradually decreased over time, indicating that the neurological function of rats in both groups gradually recovered after ischemia reperfusion. Compared with the I/R group, the neurological function improvement in the I/R + TFDM group was more significant after cerebral ischemia reperfusion. On days 7 and 14 after surgery, the mNSS scores in the I/R + TFDM group were significantly lower than those in the I/R group (Figure 2B). These results indicate that the use of TFDM can reduce the cerebral infarct volume in rats with cerebral ischemia reperfusion, and that the neurological function of such rats can be effectively improved after a period of TFDM administration.

### 3.3 TFDM alleviates neuronal damage in rats with cerebral ischemia reperfusion

**FIGURE 3:**
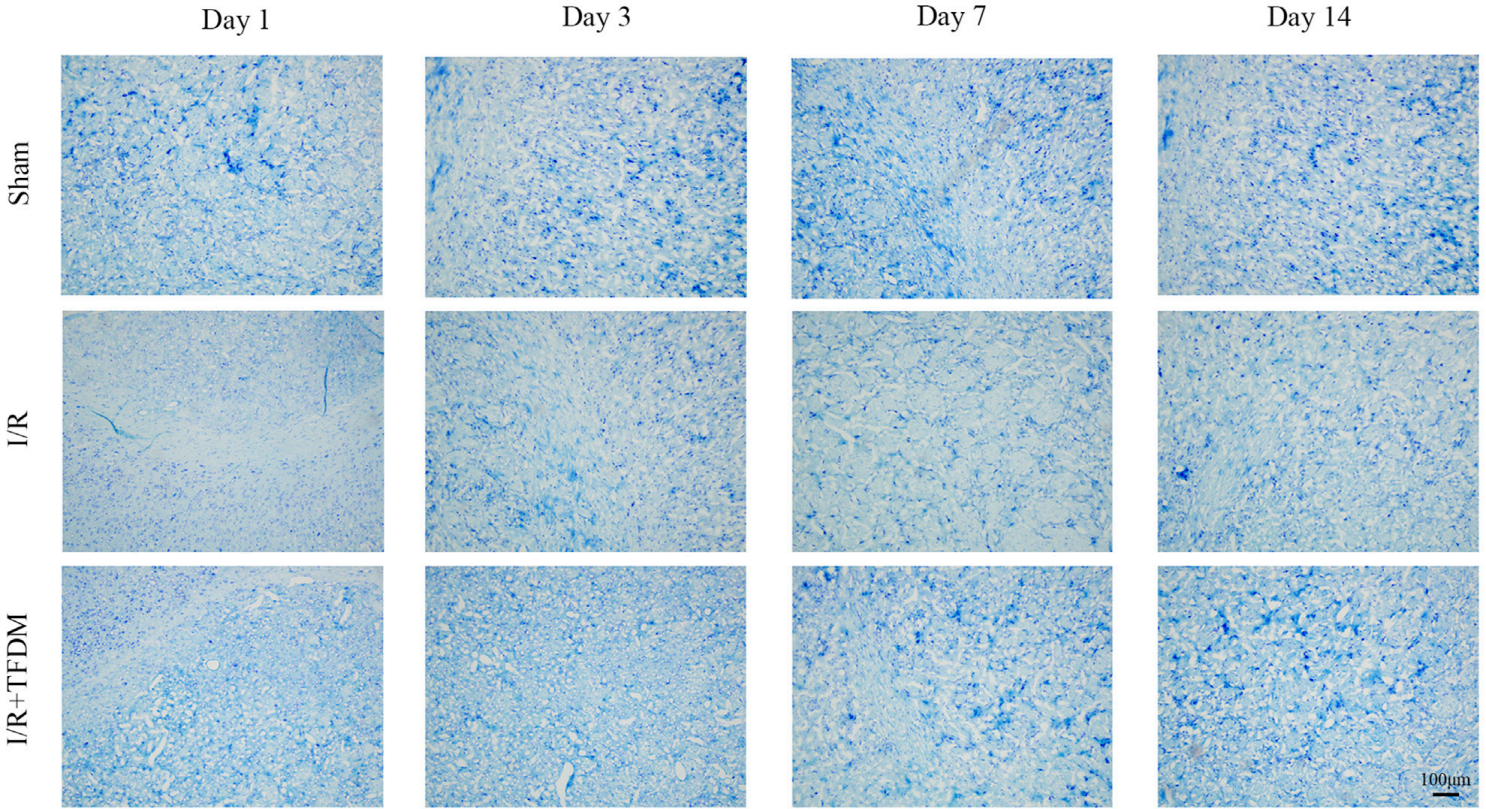
Effect of TFDM on neuronal cell structure in the cerebral ischemic semicircular band of rats after cerebral ischemia reperfusion. The tissue was stained with Nissl and observed under a 100x optical microscope.

We further investigated the effect of TFDM application on neuronal damage. The results showed that, under observation with a 100× light microscope, neurons in the brain tissue of the sham operation group exhibited blue-purple staining, with clear and regular cell boundaries and no swelling or inflammatory cell infiltration, and a large number of Nissl bodies were visible. In the I/R group, cells in the ischemic penumbra region showed disordered arrangement, obscure structure, swollen cell bodies, a reduced number of Nissl bodies, and collapse of some microvascular lumens, indicating significant neuronal damage. In the I/R + TFDM group, the disordered arrangement of cells in the ischemic penumbra region was less severe, with no obvious swelling of cell bodies, relatively mild damage, and a relatively smaller reduction in the number of Nissl bodies (Figure 3). These results indicate that the application of TFDM is effective in alleviating neuronal damage.

### 3.4 TFDM enhances blood perfusion in the ischemic penumbra of rats

**FIGURE 4:**
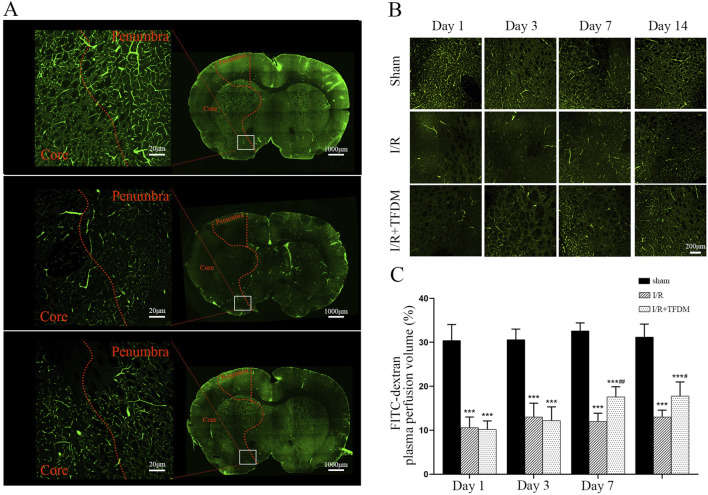
Effects of TFDM on blood perfusion and angiogenesis in cerebral ischemic penumbra of rats. **(A)** Representative micrographs of coronal sections perfused with FITC-dextran were scanned by a laser confocal microscope in whole film. From top to bottom, they were the Sham group, the I/R 7-day group, and the I/R + TFDM 7-day group. The boxed area is the range of images collected under magnification. **(B)** 100× microscopic perfusion image of ischemic penumbra. **(C)** Statistical analysis of blood perfusion in the ischemic penumbra under a 100× microscope. ^***^ *p* < 0.001 Compared with the Sham group; ^##^ *p* < 0.01, ^#^ *p* < 0.05 Compared with the I/R group.

Based on the above results, we further investigated whether TFDM affects blood supply in the ischemic penumbra using the FITC labeling method. By labeling plasma in blood vessels with FITC, we could intuitively visualize the perfusion volume and morphological characteristics of tissue blood vessels. We selected the medial region of the caudate nucleus as the main observation area (Figure 4A). Laser confocal microscopy at ×100 magnification showed that on days 7 and 14 after cerebral ischemia reperfusion, the cerebral microvascular blood perfusion in the ischemic penumbra region of the I/R + TFDM group was significantly higher than that in the I/R group (Figure 4B). In contrast, the sham operation group maintained a stable high-perfusion state at all time points after surgery (Figure 4C). The results indicated that the blood perfusion in the ischemic penumbra of the rat model was indeed enhanced after TFDM administration.

### 3.5 TFDM improves microvascular perfusion and promotes microvascular formation in the ischemic penumbra of rats

**FIGURE 5:**
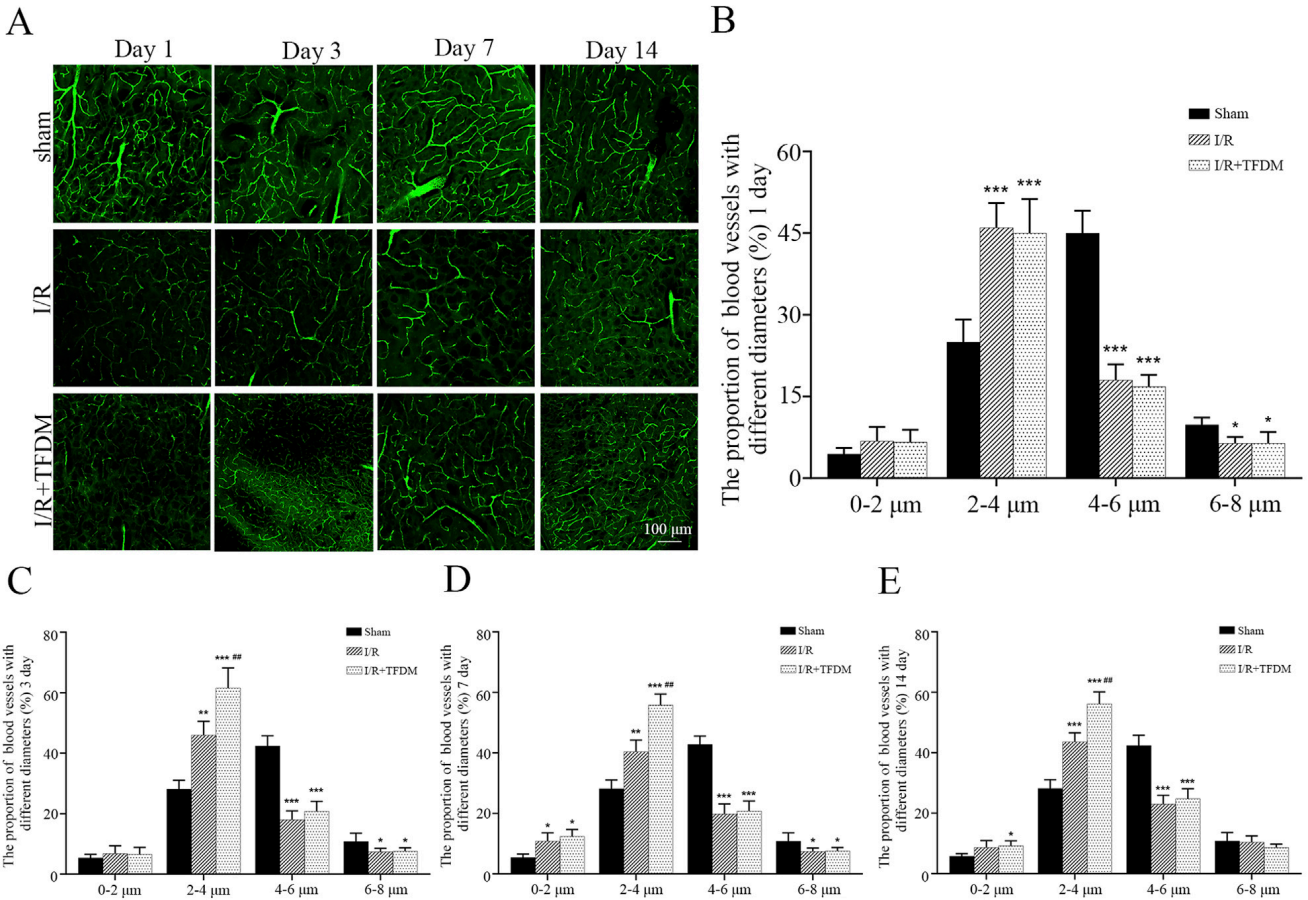
**(A)** Microscopic image of microvessels in the ischemic penumbra area at 200 times magnification. **(B–E)** Statistical analysis of microvascular diameter at different time points in the ischemic penumbra area through a 200× microscope. ^*^ *p* < 0.05, ^**^ *p* < 0.01, ^***^ *p* < 0.001 compared with the Sham group; ^##^ *p* < 0.01 compared with the I/R group.

Observation of 200× microscopic images of the ischemic penumbra showed that, compared with the sham group, the cerebral microvessels in the ischemic penumbra region of both the I/R group and the I/R + TFDM group exhibited tortuous and irregular morphologies, with an increase in the number of microvessels (Figure 5A). According to microvascular diameter analysis, on days 3 and 7 after cerebral ischemia reperfusion in rats, the proportions of blood vessels with an average diameter less than 4 μm in the ischemic side of the I/R + TFDM group were 71.6% and 63.5%, respectively, which were significantly higher than those in the I/R group (*p* < 0.01). In the sham group, the majority of blood vessels in the penumbra region at the four postoperative time points had a diameter of 4 ∼ 6 μm, accounting for 55% (Figures 5B–E). These results further indicate that TFDM improves microvascular perfusion in the cerebral ischemic penumbra of rats and promotes the formation of functional neovascularization.

### 3.6 TFDM promotes the expression of angiogenesis-related proteins VEGF, VEGFR2, and CD34 in the ischemic penumbra of rats

**FIGURE 6:**
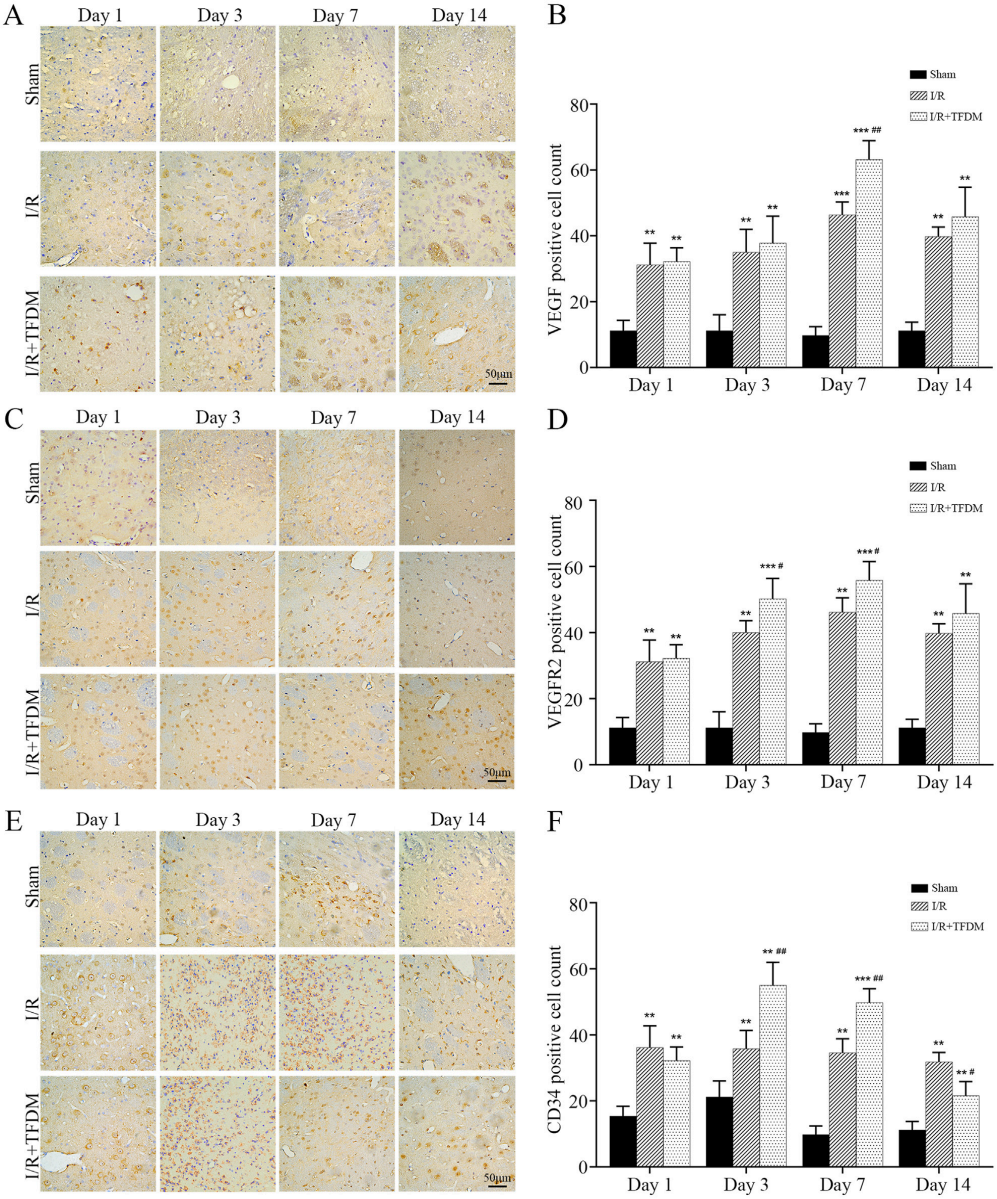
Immunohistochemical analysis of VEGF, VEGFR2, and CD34 protein expression in the penumbra region following cerebral ischemia reperfusion. **(A,C,E)** representative micrographs of VEGF, VEGFR2, and CD34 positive cells in the cerebral ischemic penumbra, detected by DAB staining; **(B,D,F)** quantitative analysis results of positive cell counts among the three experimental groups; ^**^ *p* < 0.01, ^***^ *p* < 0.001 significant compared with the sham surgery group; ^#^ *p* < 0.05, ^##^ *p* < 0.01 compared with the I/R group.

To further investigate the role of TFDM in angiogenesis within the ischemic penumbra of rat models, we detected the expression of angiogenesis-related proteins VEGF, VEGFR2, and CD34 using immunohistochemical staining. The results showed that at different time points after cerebral ischemia reperfusion surgery, the number of VEGF-, VEGFR2-, and CD34-positive cells in both the I/R group and the I/R + TFDM group was significantly higher than that in the sham group. In both the I/R group and the I/R + TFDM group, the number of VEGF and VEGFR2 positive cells in the penumbra gradually increased over time, peaking on day 7 postoperatively and then decreasing on day 14, while the number of positive cells in the sham group remained stable (Figures 6A–D). On day 7 after cerebral ischemia reperfusion, the number of VEGF-positive cells in the I/R + TFDM group was significantly higher than that in the I/R group (*p* < 0.01), with positive cells mainly concentrated in the regions adjacent to microvessels, small arteries, and small veins (Figures 6A,B). On days 3 and 7 after surgery, the number of VEGFR2-positive cells in both the I/R group and the I/R + TFDM group was significantly increased (*p* < 0.05) (Figures 6C,D). In the CD34 detection, the number of positive cells in the sham group increased slightly on days 1 and 3 after surgery, followed by a decreasing trend on days 7 and 14. However, the expression levels at all time points were significantly lower than those in the I/R group and the I/R + TFDM group. The expression levels of positive cells in both the I/R group and the I/R + TFDM group peaked on day 3 and then began to decrease. On days 3, 7, and 14 after surgery, the number of CD34-positive cells in the I/R group was significantly lower than that in the I/R + TFDM group (Figures 6E,F). These results indicate that TFDM can upregulate the expression of VEGF, VEGFR2, and CD34 in the ischemic penumbra of rats after cerebral ischemia reperfusion.

### 3.7 Western Blot analysis results further confirmed that TFDM promotes the expression of angiogenesis-related proteins VEGF, VEGFR2, and CD34 in the ischemic penumbra of rats

**FIGURE 7:**
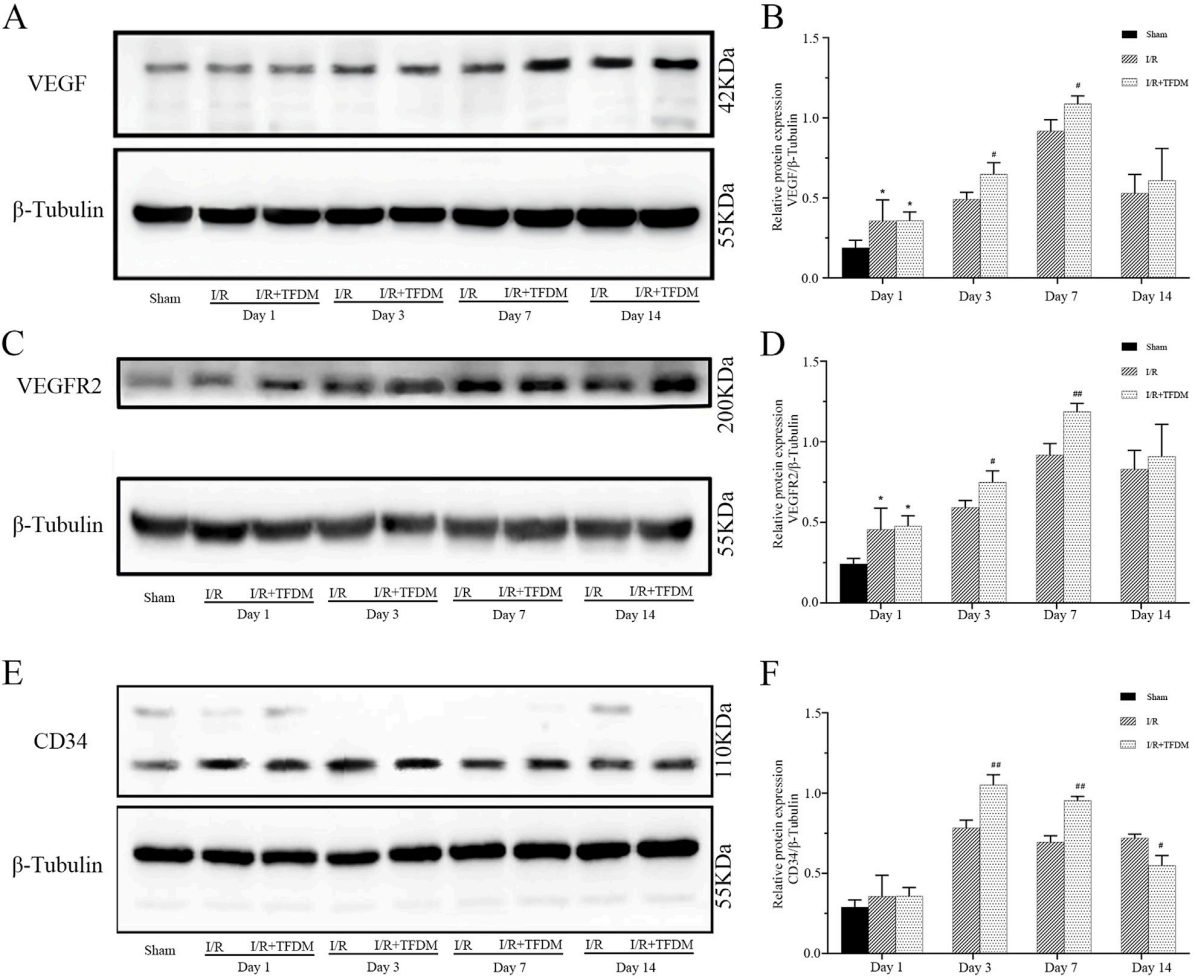
Effect of TFDM on immunohistochemically detected VEGF, VEGFR2, and CD34 proteins in the ischemic penumbra of cerebral ischemic rats. **(A,C,E)** Representative Western Blot images of VEGF, VEGFR2, and CD34 protein bands in the cerebral ischemic penumbra; **(B,D,F)** quantitative analysis of the three target protein bands (normalized to β-tubulin, internal reference protein; Sham: sham surgery group; I/R: cerebral ischemia-reperfusion group; I/R + TFDM: cerebral ischemia-reperfusion + TFDM (50 mg/kg). ^*^ *p* < 0.05 compared to the sham group, ^#^ *p* < 0.05, ^##^ *p* < 0.01 compared to the I/R group.

The results of Western Blot analysis were consistent with those of immunohistochemical observations. From days 1–14 after cerebral ischemia reperfusion in rats, compared with the sham group, the expression levels of VEGF, VEGFR2, and CD34 proteins in the ischemic penumbra of both the I/R group and the I/R + TFDM group were significantly increased. On day 7 after surgery, the expression of VEGF reached a peak. On days 3 and 7 after surgery, the expression of VEGF protein in the ischemic penumbra of the I/R + TFDM group was significantly higher than that in the I/R group at the same time points (*p* < 0.05) (Figures 7A,B). On days 3 and 7 after surgery, the expression of VEGFR2 protein in the ischemic penumbra of the I/R + TFDM group was significantly higher than that in the I/R group at the same time points (Figures 7C,D). On day 3 after surgery, the expression of CD34 in the I/R + TFDM group peaked and then began to decline (Figures 7E,F). The above experimental results indicate that TFDM promotes neovascularization by enhancing the expression of VEGF, VEGFR2, and CD34.

### 3.8 TFDM-pickering emulsion can effectively enhance the bioavailability of TFDM

**FIGURE 8:**
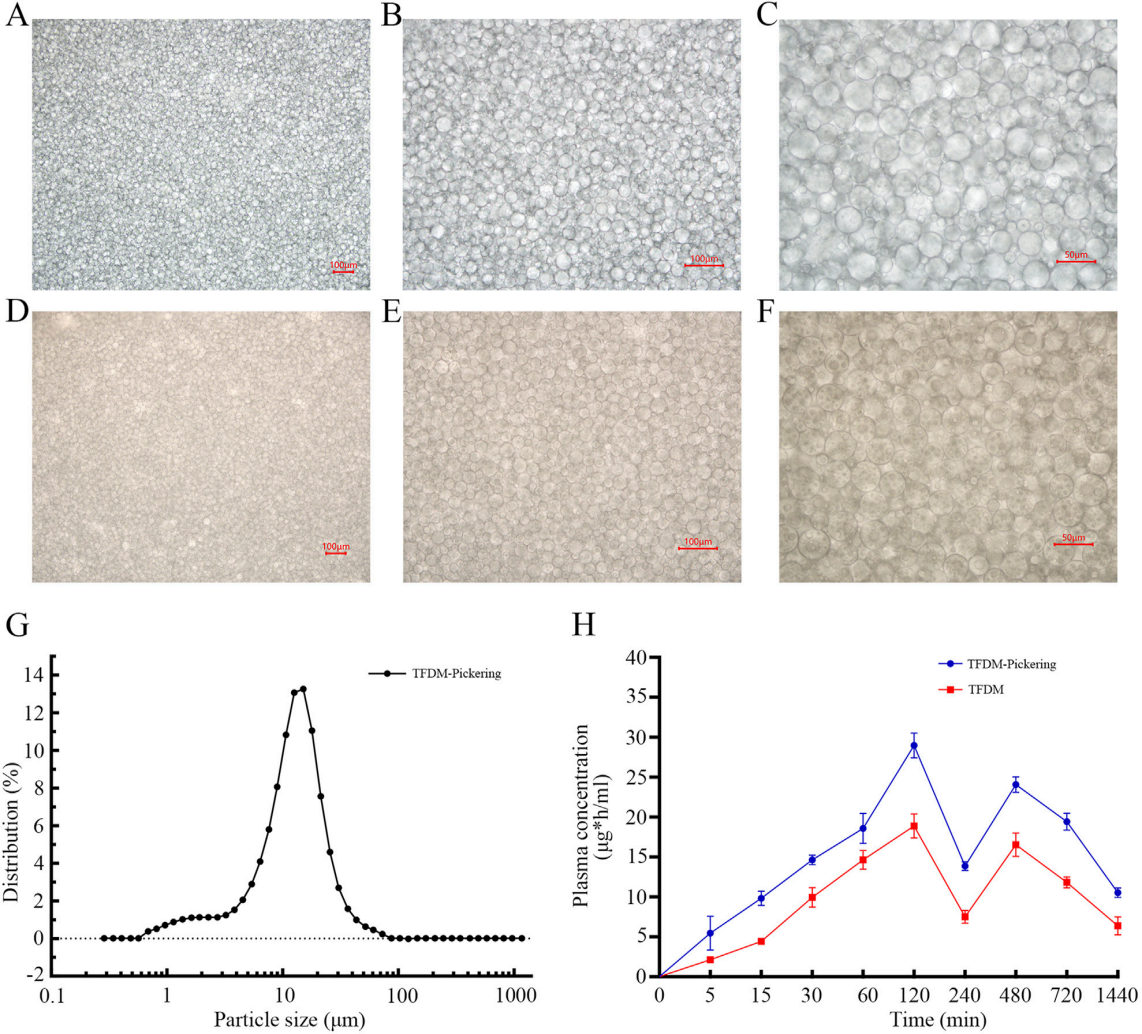
Shows microscopic images of Pickering emulsion. **(A–C)** Images of Pickering emulsion without TFDM at 100x,×200, and ×400 magnification; **(D–F)** Images of Pickering emulsion with TFDM at the same magnifications. **(G)** Particle size distribution of Pickering emulsion. **(H)** Blood concentration-time curves after oral administration of TFDM aqueous solution and TFDM-Pickering emulsion in rats.

The active components of TFDM are prone to degradation in the biological environment, and their oral bioavailability is limited. The TFDM-Pickering emulsion we designed is helpful in addressing these issues. The experimental results showed that under the microscope, the droplets of the TFDM-Pickering emulsion exhibited a more regular morphology, a more concentrated size distribution, clear droplet boundaries, and a continuous and dense particle adsorption layer was formed at the oil-water interface, with no obvious coalescence (Figures 8A–F). Most of the particle sizes of the TFDM-Pickering emulsion are around 20 μm, with a relatively uniform particle size distribution (Figure 8G). The encapsulation efficiency of TFDM in the TFDM-Pickering emulsion is 87.2%, making it an effective delivery method for TFDM. Further studies showed that there were significant differences in the plasma concentration-time curves of rats orally administered with TFDM aqueous solution and TFDM-Pickering emulsion. Both the TFDM group and the TFDM-Pickering emulsion group exhibited a bimodal phenomenon in the plasma concentration-time curves of TFDM after intragastric administration. In terms of pharmacokinetics, the TMax values of both TFDM-Pickering emulsion group and TFDM group were 120 min, and the Cmax and T1/2 values of TFDM-Pickering emulsion group were 28.98 μg/mL and 805.4 min, respectively, while those of TFDM group were 19.64 μg/mL and 730.5 min, respectively. The plasma concentration of TFDM in the TFDM-Pickering emulsion group was higher than that in the TFDM group at all time points, with the relative bioavailability of the TFDM-Pickering emulsion group being 163.55% (Figure 8H). The above results indicate that Pickering emulsion can serve as a delivery system for TFDM, and the TFDM-Pickering emulsion can effectively enhance the bioavailability of TFDM, which is beneficial for the exertion of TFDM’s biological efficacy.

## 4 Discussion

As one of the three major diseases endangering human health, stroke is an acute cerebral circulatory disorder characterized by high mortality and disability rates. Its pathological process involves complex biochemical and molecular mechanisms including excitotoxicity, calcium overload, oxidative stress, inflammatory response and apoptosis, which ultimately result in brain tissue damage, and neuronal death (Eltzschig and Eckle, 2011). Currently, therapeutic and neuroprotective options for stroke remain very limited, highlighting an urgent need to develop novel therapeutic strategies. In China, Traditional Chinese and Ethnic Medicine has a long history of being used in stroke treatment, and modern medicine research has confirmed its therapeutic potential for the disease.

In Traditional Chinese and Ethnic Medicine records, *Dracocephalum Moldavica* L. has effects of nourishing the heart, invigorating the brain, dispelling wind, relieving heat, and inducing resuscitation. Studies have shown it exerts a significant influence on angiogenesis signaling pathways and the vascular microenvironment (He et al., 2023; Zheng et al., 2020). These studies suggest it has potential for stroke treatment. Therefore, we focused on investigating its effects on cerebral vascular blood flow in stroke, applying TFDM to a cerebral ischemia-reperfusion model, and comparing the therapeutic results in detail with the model rat. TFDM was found to significantly reduce cerebral infarct volume, improve ischemic penumbra blood perfusion, alleviate ischemic area neuronal damage, and decrease rats’ mNSS. These results suggest TFDM benefits rat stroke treatment. Further analysis of rat brain tissue specimens showed that TFDM administration increased the proportion of newly formed microvessels in the cerebral ischemic region and improved local microcirculation, compared with the model rat. Tissue immunohistochemistry and Western Blot results further confirmed that TFDM-treated rats had significantly higher expression levels of VEGF, VEGFR2, and CD34 in the cerebral ischemic region than the control group. The VEGF family and its receptors are key factors in tissue angiogenesis and important targets for its regulation (Loureiro and D'Amore, 2005). In the cerebral ischemia reperfusion model, the level of VEGF is closely associated with neuroprotective effects, and increased VEGF can alleviate cerebral infarction and peripheral neuropathy (Wang et al., 2019). Besides neuroprotective effects, VEGF is also involved in neurogenesis, angiogenesis, and regeneration. VEGFR2 synergizes with VEGF-A, VEGF-C, and VEGF-D, and acts as a key factor in promoting cell proliferation, enhancing neovascularization, and boosting VEGF activity (Battaglin et al., 2018). In brain tissue, VEGFR2 directly affects neurons and Schwann cells and participates in their nutritional supply. Thus, the increased VEGF and VEGFR2 expression in TFDM-treated rat specimens strongly suggests TFDM’s potential to promote angiogenesis and exert neuroprotective effects. TFDM may be a promising strategy for stroke treatment. Additionally, CD34 is the preferred vascular endothelial cell biomarker and can indirectly reflect angiogenesis (with a positive correlation between them). Studies have shown intravenous injection of exogenous umbilical cord blood CD34^+^ cells alleviates ischemic brain injury in neonatal mice, increases nerve growth factor and VEGF expression, and reduces infarct volume (Tsuji et al., 2014). Impairment of CD34 protein function and a decrease in its expression can lead to vascular endothelial cell damage, which in turn affects blood supply to brain tissue (Krupinski et al., 2003). Our results showed that after TFDM administration, CD34 expression in the ischemia-reperfusion region of model rats peaked on day 3 and then gradually decreased over time. This aligns with the temporal pattern of post-brain injury tissue repair, possibly due to TFDM promoting CD34 expression in the ischemic region post-administration; as repair concludes, TFDM’s effect fades gradually. Thus, the above results collectively indicate TFDM has application potential for stroke treatment. Previous studies on TFDM also showed its stroke therapeutic effects mainly involve two aspects: first, activating the VEGF/tyrosine protein kinase SRC signaling pathway to promote angiogenesis; second, activating the PI3K/AKT signaling pathway to prevent cell apoptosis. This is consistent with our study results.

In our research, we identified difficulties in extracting, purifying, and preserving the active components of the traditional herb *Dracocephalum Moldavica* L. Its traditional extraction method—maceration, most commonly using water—remains in use but has very low efficiency. Given that most active components of *Dracocephalum Moldavica* L. are lipid-soluble, and ethanol—commonly used in Traditional Chinese and Ethnic Medicine extraction—is suitable for extracting diverse components (and especially effective for lipid-soluble ones), thus, we chose ethanol as the extraction solvent instead, this choice is rational. To dissolve more active components, we used low-frequency ultrasound during extraction to enhance their dissolution. We further optimized key extraction parameters: extraction temperature, extraction time, the herb-to-extractant (ethanol) volume ratio, and ethanol volume percentage. Ultimately, these improvements significantly increased TFDM extraction efficiency compared with the traditional method.

TFDM, an active component of *Dracocephalum Moldavica* L., is a flavonoid. The common issue of flavonoids—easy degradation—greatly limits their practical use in fields like medicine. To solve this issue, we subsequently selected Pickering emulsions as TFDM’s encapsulation carrier. Using solid particles to form a tightly packed adsorption layer at the oil-water interface, we enhanced TFDM’s physical stability and verified this approach’s reliability. Experimental results confirmed this Pickering emulsion improves TFDM’s oral bioavailability; the mechanism is presumably related to emulsion enhancing TFDM’s water solubility and protecting it from degradation by the gastrointestinal acidic environment and enzymes (Bao et al., 2019). Additionally, the interfacial layer formed by Pickering emulsion’s solid particles delays TFDM’s premature gastric release, enabling targeted release at intestinal absorption sites. Meanwhile, the emulsion can further disperse into small particles in the intestine, and its dispersibility can improve the absorption efficiency of intestinal epithelial cells. Thus, using Pickering emulsions as a TFDM carrier for stroke treatment constitutes a novel therapeutic regimen with significant application potential.

Traditional Chinese and natural medicines, with unique multi-targeted effects and fewer side effects, are well-suited for long-term complementary or alternative therapies. Currently, drugs targeting vascular protection and angiogenesis post-cerebral ischemia-reperfusion injury remain limited. As a vital component of Traditional Chinese and Ethnic Medicine, Mongolian Medicine has a long history, and research on its traditional prescriptions and active components for promoting angiogenesis is an active area of research. Our results show TFDM—like many Traditional Chinese and Ethnic Medicine prescriptions and their active components—effectively promotes angiogenesis in the cerebral ischemic penumbra and improves tissue microcirculation. The combination of TFDM with modern drug delivery systems enables more effective exertion of the therapeutic effects of Traditional Chinese and Ethnic Medicine, offering a new approach to stroke treatment.

## Data Availability

The original contributions presented in the study are included in the article/supplementary material, further inquiries can be directed to the corresponding authors.
